# A New Technique of Heterotopic Heart Transplantation with Direct Pulmonary Artery Anastomosis

**Published:** 2017-10

**Authors:** Prashant Vaijyanath

## Introduction

**Figure 1:**
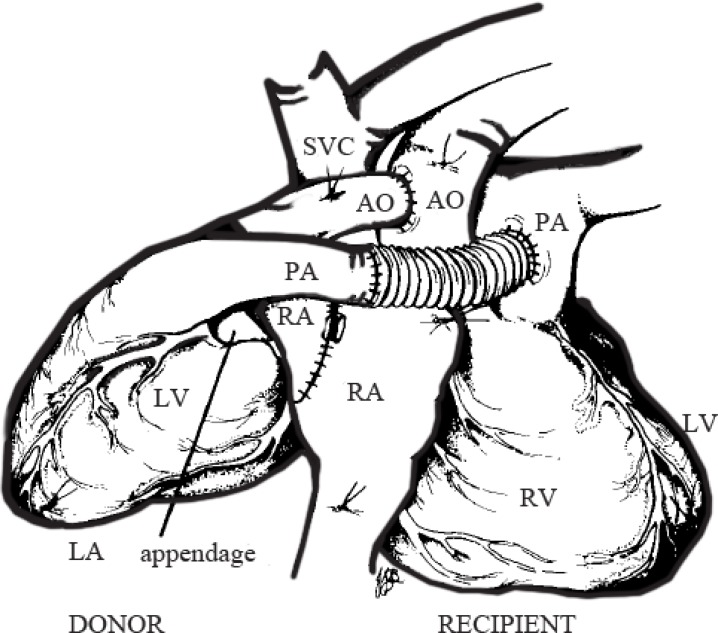
Current technique of heterotopic heart transplantation with an interposition conduit between the donor and recipient pulmonary artery. AO, Aorta; IVC, Inferior vena cava; LA, Left atrium; LPA, Left pulmonary artery; LPV, Left pulmonary vein; RA, Right atrium; RPA, Right pulmonary artery; RPV, Right pulmonary vein; SVC, Superior vena cava

The current technique for heterotopic cardiac transplantation in patients with high pulmonary vascular resistance entails the interposition of a Dacron conduit^1^ between the native and donor pulmonary artery (PA) as it is not possible for the donor PA to reach the recipient PA (Figure 1). The original technique of Bernard and Losman^2^ was abandoned due to intractable arrhythmias. Da Silva et al.^3^ proposed an anastomosis between the donor main PA and the inferior margin of the recipient right PA.

We describe a technique of a direct PA anastomosis between the anterior surface of the recipient right PA and the donor left PA an in end-to-side fashion after closing the donor right PA with suture. We believe that this technique confers an excellent anatomic orientation, thereby reducing the chances of the kinking and torsion of the anastomosis.

***Surgical Technique***

We used this technique in a 48-year-old male patient of end-stage ischemic cardiomyopathy with prior multiple percutaneous coronary interventions and cardiac resynchronization therapies. The calculated pulmonary vascular resistance was 7.1 WU and the systolic PA pressure was 82 mmHg with a systemic pressure of 95/50. The patient’s severe pulmonary hypertension precluded orthotopic transplantation.

***Donor Heart Preparation***

After standard donor cardiectomy, the ostium of the right pulmonary veins and the right PA was closed. The inferior vena cava (IVC) was not closed, and nor was a posterior opening made in the superior vena cava (SVC)-right atrium junction of the donor heart for a cavo-atrial anastomosis.

***Recipient Operation***

**Figure 2:**
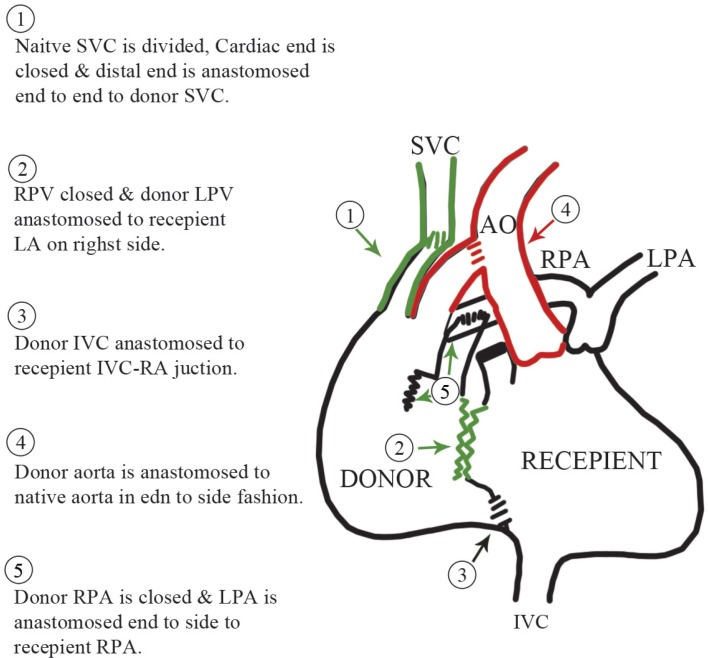
A new technique of heterotopic heart transplantation with direct pulmonary artery anastomosis. AO, Aorta; IVC, Inferior vena cava; LA, Left atrium; LPA, Left pulmonary artery; LPV, Left pulmonary vein; RA, Right atrium; RPA, Right pulmonary artery; RPV, Right pulmonary vein; SVC, Superior vena cava

After a standard cardiopulmonary bypass, right-sided pleuro-pericardial reflection was divided to create space for the donor heart in the right pleural cavity. The recipient SVC was transected near the right atrium, its lower stump was sutured, and its upper stump was laterally mobilized, which exposed the right PA. A left atrial incision was made medially to the right superior pulmonary vein and extended in the interatrial groove. The donor heart was placed in the right side of the chest, and the 2 left atria were sutured in a position that allowed the donor left PA to easily reach the recipient right PA. The anterior surface of the recipient right PA was opened and a cobra hood posterior slit was made on the donor left PA, almost extending to the main PA, and a direct anastomosis was achieved without tension. This was followed by an end-to-end donor/recipient SVC anastomosis and then an end-to-side anastomosis of the donor IVC to the recipient IVC as low as possible, allowing the lower body venous return of the recipient to reach the donor right atrium. The donor ascending aorta, which is usually long, was anastomosed as distally as possible in the recipient ascending aorta to create space and access to the PA anastomosis in case of bleeding (Figure 2).

The patient was easily weaned off cardiopulmonary bypass with good hemodynamic and functional recovery. The patient’s systolic PA pressure had fallen to half systemic arterial pressure by the time he left the intensive care unit.

## Discussion

We believe that the method herein expounded has certain merits. The technique can prevent the theoretic complications of a PA prosthetic graft such as compression, infection, thrombosis, fibrosis, and difficulty in sternal closure. Moreover, achieving a bicaval anastomosis seems to have dual advantages. The first advantage on the SVC side is that the closure of the recipient cardiac end unloads the dysfunctional native heart and endomyocardial biopsy is facilitated because the SVC anastomosis leads the bioptome forceps easily to the donor right ventricle. The other positive point on the IVC side is the sharing of lower body blood and burden between the recipient and donor heart.
